# [Retracted] Inhibiting proliferation and migration of lung cancer using small interfering RNA targeting on Aldo‑keto reductase family 1 member B10

**DOI:** 10.3892/mmr.2023.13109

**Published:** 2023-10-02

**Authors:** Zhen Zhou, Yi Zhao, Lingping Gu, Xiaoming Niu, Shun Lu

Mol Med Rep 17: 2153–2160, 2018; DOI: 10.3892/mmr.2017.8173

Following the publication of this paper, it was drawn to the Editor's attention by a concerned reader that data from the cell adhesion experiment shown in Fig. 11 were strikingly similar to data appearing in different form in Fig. 6B in another article written by different authors at different research institutes [Huang Z, Li J, Du S, Tang Y, Huang L, Xiao L and Tong P: FKBP14 overexpression contributes to osteosarcoma carcinogenesis and indicates poor survival outcome. Oncotarget 7: 39872-39884, 2016].

Owing to the fact that the contentious data in the above article had already been published prior to its submission to *Molecular Medicine Reports*, the Editor has decided that this paper should be retracted from the Journal. The authors were asked for an explanation to account for these concerns, but the Editorial Office did not receive a reply. The Editor apologizes to the readership for any inconvenience caused.

